# 

**Published:** 1855-07

**Authors:** 


### CONTENTS OF NO. V., VOL. II.

ORIGINAL COMMUNICATIONS.

Cases in Practice—By Prof. McGugin . . . . 321
Operation for Imperforate Anus—By Prof. Hughes . . 330
Suggestions as to the Method of Using Narcotics in Nervous Diseases—By John R. Allen, M. D. . . . 332

DEPARTMENT OF SELECTIONS.

Practical Observations on Puerperal Fever—By L. Shanks, M. D. . . . . . . . . 337
The Nature of Nursing Sore Mouth—By M. L. Knapp, M.D. 349
Letter to Dr. Bowling—By Emma Willard . . . 361
Account of the Cholera in Columbia—By B. T. Heber Jackson, M. D. . . . . . . . . 364
Affections of the Cervix Uteri—By Luther Parks, M. D. 374
Extracts from Records of Boston Society for Medical Improvement . . . . . . . 381

EDITORIAL DEPARTMENT.

Eighth Annual Meeting of the American Medical Association . . . . . . . . 365
Prof. John R. Allen, M. D. . . . . . . 343
College Announcements . . . . . . 344
Iowa Medical Society . . . . . . . 346 396
Medical Society in Wapello County . . . . 348
A Circular . . . . . . . . 348
Our Journal . . . . . . . . 349
Prof. Austin Flint . . . . . . . 350

